# Associations between e-health literacy and chronic disease self-management in older Chinese patients with chronic non-communicable diseases: a mediation analysis

**DOI:** 10.1186/s12889-022-14695-4

**Published:** 2022-11-29

**Authors:** Ying Wu, Jing Wen, Xiaohui Wang, Qingyao Wang, Wen Wang, Xiangjia Wang, Jiang Xie, Li Cong

**Affiliations:** 1 School of Medicine, Hunan Normal University, Changsha, 410013 People’s Republic of China; 2 Xiangya Hospital, Central South University, Changsha, 41008 People’s Republic of China

**Keywords:** Chronic non-communicable diseases, Self-management, E-health literacy, Self-efficacy, Mediation analysis

## Abstract

**Background:**

Chronic non-communicable diseases (CNCDs) are an urgent public health issue in China, especially among older adults. Hence, self-management is crucial for disease progression and treatment. Electronic health (e-health) literacy and self-efficacy positively correlate with self-management. However, we know little about their underlying mechanisms in older adults with CNCDs.

**Objective:**

To explore the factors that influence chronic disease self-management (CDSM) and verify self-efficacy as the mediator between e-health literacy and self-management behavior in older patients with CNCDs.

**Methods:**

This cross-sectional study included 289 older patients with CNCDs from Hunan province, China, between July and November 2021. E-health literacy, self-efficacy, social support, and CDSM data were collected through questionnaires. The influence of each factor on CDSM was explored with multiple linear regression analysis. Intermediary effects were computed via a structural equation model.

**Results:**

The total CDSM score in the patients was 29.39 ± 9.60 and only 46 (15.92%) patients used smart healthcare devices. The regression analysis showed e-health literacy, self-efficacy, and social support were the factors that affected CDSM. Furthermore, the structural equation model revealed that self-efficacy directly affected CDSM (*β* = 0.45, *P* < 0.01), whereas e-health literacy affected it directly (*β* = 0.42, *P* < 0.01) and indirectly (*β* = 0.429, *P* < 0.01) through self-efficacy.

**Conclusions:**

This study revealed that self-management among older patients with CNCDs is at a low level, and few of them use smart healthcare devices. Self-efficacy plays a partial intermediary role between e-health literacy and self-management in older patients with CNCDs. Thus, efforts to improve their CDSM by targeting e-health literacy may be more effective when considering self-efficacy.

**Supplementary Information:**

The online version contains supplementary material available at 10.1186/s12889-022-14695-4.

## Introduction

Chronic non-communicable diseases (CNCDs) impose an enormous economic burden on patients, their families, and society due to their high morbidity, high mortality, low control rate, and low awareness, especially for older patients [1, 2]. According to the Global Burden of Disease Study, an estimated 41.1 million (73.4%) of the 55.9 million deaths worldwide were from CNCDs in 2017, and this figure may reach 52 million by 2030 [3]. Currently, China has 260 million patients with confirmed chronic disease, with an average annual growth rate of 8.9%, accounting for 88.5% of the total deaths [4]. Thus, chronic diseases of older adults are a serious public health issue, and their management is a global challenge. Self-management is a set of approaches an individual uses daily to manage their chronic condition and promote recovery. Efficient self-management of older patients with chronic diseases may improve disease prognosis and life quality, optimizing the allocation of healthcare resources.

Several chronic disease management theoretical models have been implemented internationally, and the two most representative are the Chronic Care Model (CCM) [5] and the Innovative Care for Chronic Conditions (ICCC) Framework [6]. CCM suggests that providing high-quality care for chronic diseases depends on the coordination of several essential components: clinical information systems, decision support, delivery system design, self-management support, and community resources [5]. ICCC Framework is an expanded version of CCM proposed by World Health Organization (WHO) in 2002. It was developed to suit the limited health resources of low-income countries and emphasizes the importance of community leaders and caregivers in providing efficient care [6]. However, implementing either model is challenging because each requires a complete healthcare system, a well-developed medical organization, and professional medical staff [7]. Chronic disease self-management (CDSM) involves daily strategies an individual uses to control their diseases [8], such as exercise, diet, health checkups, and medication. Interventions targeting self-management are effective and affordable, gaining worldwide attention [9, 10]. Therefore, instead of implementing financially constrained models and medically resource-constrained models, CDSM can be used to improve the health status and quality of life and reduce the incidence of complications in older adults with CNCDs [11, 12].

In China, the self-management of older patients with CNCDs is still at low levels [13]. Most have limited knowledge of their disease, and its mismanagement may result in additional complications and medical costs. In addition, prolonged physical discomfort and treatment reduce psychological well-being and quality of life, leading to anxiety, depression, and other mental disorders. Older patients with CNCDs rely on social support from their healthcare providers, families, and caregivers to make decisions and adjust their self-managed health behaviors [14]. Self-efficacy is the expectation and confidence in achieving specific goals, which is a vital mediator between CDSM and health behaviors [15]. A stronger sense of self-efficacy determines adherence to health-related behaviors and may be improved by developing e-health literacy [16].

E-health literacy is the ability of individuals to obtain, understand, and evaluate health information through online electronic media [17]. Higher e-health literacy in seniors promotes using various smart medical devices for disease risk self-assessment and monitoring physical status to improve their CDSM [18]. Van et al. [19] reported that e-health literacy enhances CDSM to make better judgments for maintaining a healthy status. However, we know little about the potential relationship between e-health literacy, self-efficacy, and CDSM. Moreover, the mediating role of self-efficacy between e-health literacy and CDSM is elusive. Thus, we wondered whether e-health literacy facilitates CDSM through self-efficacy.

This study aimed to explore the application of smart healthcare products and self-management status in older patients with CNCDs in China. It developed a structural equation model to assess the factors influencing CDSM and distinguish between direct and indirect effects. The results provide new insights for improving chronic disease self-management of older patients through e-health literacy and self-efficacy.

## Methods

### Study design and participants

This cross-sectional study randomly selected 300 older patients with CNCDs from 4 nursing homes in Hunan province, China, from July to November 2021. Those included satisfied the following criteria: (1) Were 60-year-olds or older; (2) Had at least one CNCD that meets the WHO diagnostic criteria [20]; (3) Were literate and understood the questionnaires; (4) Volunteered to participate. Patients with mental disorders, communication impairment, or serious illnesses and complications were excluded. We informed the patients of the aim, content, investigation procedures, and the possibility of withdrawal from the study at any time. All eligible participants gave written informed consent before starting the survey, and completed pen-and-paper surveys in separate quiet rooms. The assessors were uniformly trained according to the study protocol. They contacted participants and assisted those who had difficulties reading. The entire survey took approximately 15 min and was anonymous and voluntary. The completed questionnaires were checked individually to evaluate data quality and integrity. Of the 300 elderly patients invited, 8 declined to participate due to health problems or lack of interest, and 3 were interrupted during the survey. Finally, 289 valid questionnaires were returned, with a 96.33% response rate. The Ethics Committee of Hunan Normal University (No. 2021291) approved the study.

### Instruments

#### General information questionnaire

The general information questionnaire collected sociodemographic and health-related information of older patients with CNCDs. The sociodemographic data involved gender, age, number of children, marital status, educational status, occupation, living situation, main caregiver, residence time in Changsha (in years), and monthly household income (RMB). The health-related information covered the history of hospitalization, family history, years with CNCDs, number of CNCDs, frequency of medication, and use of smart healthcare devices.

#### Chronic disease self-management measurements

Self-management status was measured with a generic scale established in 1999 at the Patient Education Research Center, Stanford University. It evaluates the effect of implementing chronic disease self-management programs [21]. The Chinese version [22] assessed self-management behavior of older patients with CNCDs by measuring the following dimensions: 1) Exercise or the time patients spent doing aerobic, anaerobic, weight training, and other exercises in the past week (6 items); 2) Communication skills or whether patients ask their doctors about disease-related treatments and express disease-related worries (3 items); 3) Cognitive disease management or the treatment of choice for elderly patients when they experience physical discomfort (6 items). Each item was measured with a 5-point Likert scale between 1 (never) and 5 (very frequently). Total scores ranged from 15 to 75, with higher indicating higher self-management. The Chinese version had high sensitivity and validity in older patients with CNCDs, Cronbach’s α, measuring internal consistency, ranged between 0.79 and 0.85 [22].

#### E-health literacy scale

E-health literacy was measured using an e-health literacy scale developed by Norman and Skinner in 2006 to assess a population’s ability to use information technology and adjust health behavior [23]. The Chinese version [24] measured 3 dimensions in older patients with CNCDs: Application ability or the ability to go online and find health information (5 items); Discrimination capability or the ability to distinguish online information relevant to their condition (2 items); Decision-making capability or the confidence to make health-related decisions based on the relevant information (1 item). Each item was measured with a 5-point Likert scale from 1 (totally disagree) to 5 (totally agree). Total scores ranged from 8 to 40, with higher scores reflecting higher e-health literacy. The Chinese version showed good reliability among older patients in the community, Cronbach’s α for internal consistency was 0.98 [24].

#### General self-efficacy scale

The general self-efficacy scale was developed by Jerusalem and Schwarzer in 1979 to measure an individual’s self-confidence in coping with challenges in various environments [25]. The scale, containing 10 questions, tested the confidence to face difficulties and the ability to stick to ideals and reach goals in older patients with CNCDs. The scale was rated on a 4-point Likert scale from 1 (not at all true) to 4 (exactly true). The total score was between 10 and 40, with a higher score representing greater self-efficacy. The reliability and validity were demonstrated in 26 countries [26], and the Chinese version had high sensitivity and validity. Cronbach’s α for internal consistency was 0.85 [27].

#### Social support self-rating scale

The social support self-rating scale was developed in 1994 by Xiao to determine the type and level of received social support [28]. It measured 3 dimensions of an older patient with CNCDs received with social relationships, with 10 items: 1) Objective support or actual support the patient received (3 items). 2) Subjective support or emotional experience of being respected, supported, and understood (4 items). 3) Utilization of support or the patient’s use of distinct types of social supports, including confiding in, asking for help, and participating in activities (3 items). Each subjective support and utilization of support entry was rated on a 4-point Likert scale. Objective support was measured according to the number of social support sources. The total score ranged from 10 to 66, with higher scores indicating higher levels of social support. The scale showed reasonable internal consistency (Cronbach’s α = 0.89) and test-retest reliability (Cronbach’s α = 0.92) [29].

#### Smart healthcare devices questionnaire

This scale was developed by researchers based on the System Usability Scale (SUS) and the user version of the Mobile Application Rating Scale (uMARS). SUS was used to measure the user’s experience of effectively performing tasks in various health products with 10 items. Each item was measured on a 5-point Likert scale ranging from 1 (strongly disagree) to 5 (strongly agree). The odd-numbered items were scored using “Original Score-1” and the even-numbered using “5-Original Score.” The conversion scores for all items were added together and multiplied by 2.5 to obtain total scores between 0 and 100. Cronbach’s α for internal consistency was 0.851. Higher scores reflect better usability [30]. The uMARS encompasses 6 items to assess the perceived impact of a mobile application on the users’ awareness, knowledge, attitudes, willingness to change, and actual change of the target health behavior. All items were rated on a 5-point scale from 1 (strongly disagree) to 5 (strongly agree). The total uMARS scores ranged from 5 to 30, and a higher score represented a better valuation. Cronbach’s α for internal consistency was 0.890 [31]. Product scores are scored by patients based on their own experience of using the device, with a total score of five. The scale also includes general information, product functions, price, security, quality, and cost performance.

### Statistical analysis

Statistics were calculated using SPSS version 22.0 and AMOS version 23.0 (IBM Corp, Armonk, NY). Categorical data were shown as numbers and percentages, while continuous data as the mean ± standard deviation (SD). The univariable analysis (t-test or ANOVA test, as appropriate) investigated the association between the general patient information (sociodemographic characteristics and health-related information) and the main study variables (CDSM, e-health literacy, self-efficacy, and social support), as well as the associations between smart medical products and the product, SUS, uMARS scores. Pearson’s correlation analysis, multiple stepwise linear regression analysis, and structural equation modeling determined the structural relationship among the main variables. A bootstrap resampling technique was employed to evaluate the significance of mediating effect. The final mediating effect percentage is equal to the indirect effect score divided by the total effect score. The model fit was assessed using the following model-fit indices [32]: chi-square (χ^2^), goodness of fit index (GFI), adjusted goodness-of-fit index (AGFI), incremental fit index (IFI), comparative fit index (CFI), Tucker-Lewis index (TLI), normed fit index (NFI), root mean square error of approximation (RMSEA). Statistical significance was inferred when *P* < 0.05.

## Results

### Participant characteristics, mean scores, and correlational analyses of study variables

We included a total of 289 patients with CNCDs in this study. Their mean age was 68.61 ± 5.36 years, ranging from 60 to 86 years. Demographic characteristics of older patients participating in the study are shown in Table 1. Total and mean scores of CDSM, e-health literacy, self-efficacy, social support, and each dimension are shown in Table 2. Pearson’s correlation analysis revealed a significant positive correlation between the total scores of e-health literacy, self-efficacy, and social support and CDSM and its 3 dimensions (*r* = 0.147 to 0.408; *P* < 0.01, Table 2). More information on the relationship between demographic characteristics and the study variables is provided in Table S1.

**Table 1:** Demographic characteristics of older patients with chronic non-communicable diseases (*n* = 289)

Variable	N	%
Gender		
Male	181	62.6
Female	108	37.4
Age (years)		
60–70	185	64
71–80	97	33.6
81–90	7	2.4
Marital status		
Married	182	63
Single	62	21.5
Widowed	36	12.5
Divorced	9	3.1
Education status		
Primary school	97	33.6
Junior school	96	33.2
High school	66	22.8
University	30	10.4
Hospitalization history		
Yes	67	23.2
No	222	76.8
Years of CNCDs		
< 5	60	20.8
5–10	85	29.4
> 10	144	49.8
Number of CNCDs		
1	63	21.8
2	134	46.4
≥ 3	92	31.8

*CNCDs* Chronic non-communicable diseases

**Table 2:** Mean scores and correlation matrix of main study variables (*n* = 289)

Variable	Mean (SD)	CDSM	E-health literacy	Self-efficacy	Social support
CDSM	29.39 (9.60)	1	0.359**	0.340**	0.222**
Exercise	5.66 (3.54)	–	0.302**	0.352**	0.267**
Cognitive management	16.14 (4.71)	–	0.336**	0.352**	0.223**
Connect with doctors	7.58 (3.44)	–	0.408**	0.147**	0.235**
E-health literacy	19.15 (9.60)	0.359**	1	0.346**	0.210**
Application	12.26 (6.55)	0.395**	–	0.308**	0.193**
Identification	4.59 (2.29)	0.371**	–	0.360**	0.233**
Decision-making	2.28 (1.32)	0.359**	–	0.350**	0.199**
Self-efficacy	24.74 (6.51)	0.340**	0.346**	1	0.369**
Social support	42.25 (6.29)	0.222**	0.210**	0.369**	1
Objective support	10.37 (2.96)	0.401**	0.233**	0.242**	–
Subjective support	24.60 (4.21)	−0.113**	0.251**	0.280**	–
Support usage	7.28 (2.29)	0.336**	0.256**	0.161**	–

*CDSM* Chronic disease self-management, *SD* Standard deviation. **P < 0.01

### Univariable analysis of chronic disease self-management, e-health literacy, self-efficacy, and social support

Univariable analyses showed the CDSM score increased for older patients with the following sociodemographic and health-related information: 70 years or younger, married, self-employed, live with spouse, living in Changsha for 10 years or more, monthly income higher than 2000 RMB, no hospitalization history, less than 5 years of CNCDs, only one CNCD, use smart healthcare devices (*P* < 0.05). Results for other study variables (e-health literacy, self-efficacy, and social support) and detailed data are shown in Table S1.

### Multiple linear regression analysis of chronic disease self-management

A multiple linear regression model was constructed using CDSM as the dependent variable. E-health literacy, self-efficacy, social support with its 3 dimensions, and the significant variables in the univariable analysis (age, marital status, occupation status, residence status, monthly income, annual hospitalization history, disease duration, number of CNCDs, and use of smart healthcare devices) were used as the independent variables. E-health literacy (*β* = 0.308, *P* < 0.01), self-efficacy (*β* = 0.574, *P <* 0.01), objective support (*β* = 0.762, *P <* 0.01), and support usage (*β* = 0.825, *P <* 0.01) were positively correlated with CDSM. Conversely, subjective support was negatively associated with CDSM (*β* = − 0.846, *P <* 0.01). Detailed results of the regression analysis are shown in Table 3.

**Table 3:** Multiple linear regression analysis of factors influencing CDSM in older patients with CNCDs (*n* = 289)

	B	SE	*β*	*t*	*P-value*
Constant	16.199	2.447		6.619	*P <* 0.01
E-health literacy	0.308	0.042	0.309	7.302	*P <* 0.01
Self-efficacy	0.574	0.067	0.389	8.591	*P <* 0.01
Objective support	0.762	0.132	0.235	5.753	*P <* 0.01
Support usage	0.825	0.171	0.196	4.808	*P <* 0.01
Subjective support	−0.846	0.092	−0.372	−9.153	*P <* 0.01

*SE* Standard error

### Construction and testing of structural equation model

**Fig. 1:**
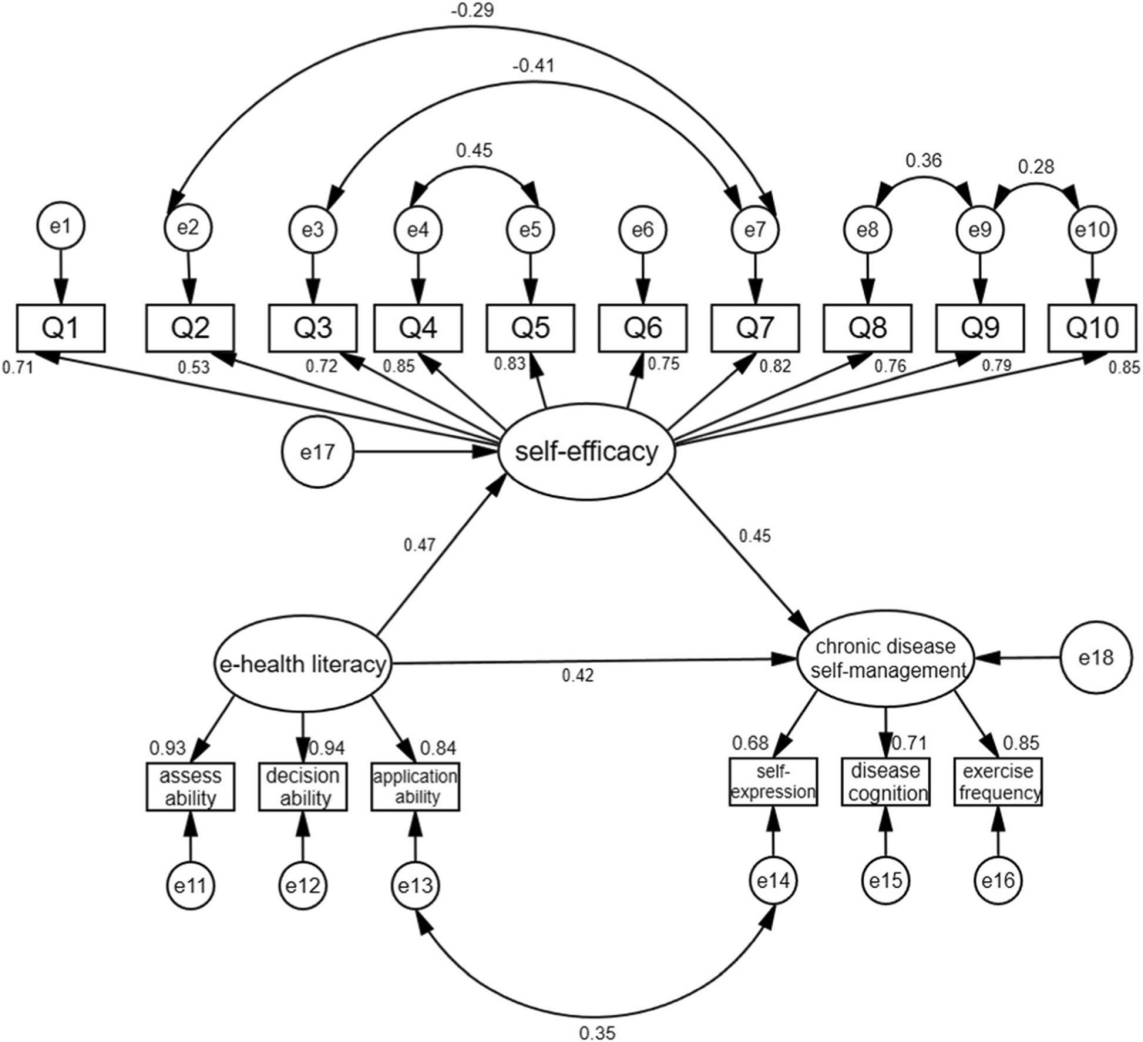
Fitting model of mediation of self-efficacy among the elderly with CNCDs (with standardized regression coefficients). Q1–10: Questions 1 to 10, e1–10: error 1–10

Structural equation modeling was performed to assess the path relationship between CDSM, e-health literacy, and self-efficacy. The parameters were estimated using the maximum likelihood method, and various model fit indices evaluated the fitting of the theoretical model and data. Model-fit indices (χ^2^/df = 2.662; GFI = 0.935; AGFI = 0.908; IFI = 0.961; CFI = 0.961; TLI = 0.945; NFI = 0.940; RMSEA = 0.076) indicated good model fit. The model results showed that e-health literacy and self-efficacy positively predicted CDSM (*β* = 0.42, *β* = 0.45, *P <* 0.001), supporting the predictions of our regression analysis. Furthermore, e-health literacy positively predicted self-efficacy (*β* = 0.47, *P <* 0.001). The mediating effect of self-efficacy between e-health literacy and CDSM was 0.429. The total effect of e-health literacy on CDSM was 1.278, of which 33.56% constituted the mediating effect. All paths are shown in Fig. 1. Standardized direct, indirect, and mediated effects are summarized in Table 4.

**Table 4:** Direct, indirect, and total effects of predictor variables in the model

	Pathway	Value	SE	95% CI	Proportion
Direct effect	e-health literacy to CDSM	0.849	2.405	0.508–1.180	66.43%
Mediated effect	self-efficacy between e-health literacy and CDSM	0.429	0.034	0.960–1.642	33.56%
Total effect	e-health literacy to CDSM	1.278	2.805	0.269–0.606	

*CI* Confidence interval, *CDSM* Chronic disease self-management

### Univariate analysis of smart healthcare devices on the product, SUS, and uMARS scores

Only 46 (15.92%) participants in this study used smart healthcare devices. Overall, those who used the devices had higher CDSM, e-health literacy, self-efficacy, and social support than those who did not (*P* < 0.05, Table S1). Product, SUS, and uMARS scores were higher for devices recommended by medical staff (*P* < 0.05). In addition, product scores and uMARS scores were higher for patients highly likely to recommend the devices (*P* < 0.05). The cost-effective and good-quality medical products also had better uMARS scores (*P* < 0.05). Detailed results of the analysis are shown in Table 5.

**Table 5:** Associations of smart healthcare products with the product, SUS, and uMARS scores (*n* = 46)

Variable	N	%	Product scores		SUS scores		uMARS scores	
			$\overline{x}$ ± s	t/F，*P*	$\overline{x}$ ± s	t/F，*P*	$\overline{x}$ ± s	t/F，*P*
Information Sources								
Online media	8	17.2	4.04 ± 0.55	F = 3.007	80.00 ± 14.90	F = 9.383	21.40 ± 2.88	F = 4.113
Medical staff	20	43.5	4.40 ± 0.68	*P =* 0.041	85.25 ± 6.73	*P <* 0.001	23.30 ± 3.32	*P =* 0.012
Friends	18	39.1	3.61 ± 1.13		82.50 ± 13.96		18.83 ± 4.79	
Recommend intention								
Highly	38	82.6	4.34 ± 0.67	t = −6.559	84.21 ± 9.08	t = −1.126	22.13 ± 3.21	t = −3.325
General	8	17.4	2.63 ± 0.69	*P <* 0.001	79.38 ± 18.16	*P =* 0.266	17.13 ± 6.31	*P =* 0.002
Product quality								
Good	39	84.8	4.18 ± 0.78	F = 3.007	85.19 ± 8.69	F = 0.703	21.95 ± 3.42	F = 5.649
Average	7	15.2	3.33 ± 1.51	*P =* 0.060	74.17 ± 18.75	*P =* 0.501	16.33 ± 6.47	*P =* 0.007
Cost performance								
High	29	63.0	4.22 ± 0.82	t = −1.749	83.53 ± 9.58	t = −0.131	23.28 ± 2.52	t = −5.262
Low	17	37.0	3.74 ± 1.06	*P =* 0.087	83.09 ± 13.56	*P =* 0.897	17.82 ± 4.53	*P <* 0.001
Product price (RMB)								
0–1000	34	73.9	3.91 ± 0.95	t = 1.636	75.36 ± 10.76	t = 1.954	20.29 ± 4.46	t = 2.761
≥ 1000	12	26.1	4.42 ± 0.79	*P =* 0.109	81.87 ± 6.75	*P =* 0.057	24.00 ± 2.00	*P <* 0.001
Product functions								
Monitoring	32	69.6	3.94 ± 1.01	F = 0.607	75.55 ± 11.34	F = 0.794	20.41 ± 4.51	F = 2.358
Behavior management	4	8.7	4.00 ± 0.816	*P =* 0.614	79.17 ± 11.81	*P =* 0.254	21.75 ± 4.92	*P =* 0.085
Synthesis	10	21.7	4.40 ± 0.69		80.75 ± 4.57		23.80 ± 1.93	

$\overline{x}$ ± s: Mean ± standard deviation, *SUS* System usability scale, *uMARS* User version of the mobile application rating scale

## Discussion

This study provided evidence of the relationship between e-health literacy, self-efficacy, social support, and self-management among older adults with CNCDs. Hence, it deepens our understanding of the factors that improve self-management in an older population. The structural equation model supported our hypothesis that e-health literacy and self-efficacy are crucial predictors of self-management in older patients with CNCDs. In addition, e-health literacy indirectly influenced self-management through self-efficacy, confirming the mediatory role.

Our findings showed that older people with CNCDs have lower levels of CDSM, revealing similarities and differences with existing studies [11, 33]. For example, our univariable analysis showed that older adults with multiple chronic diseases and longer disease duration had lower levels of self-management. Indeed, prolonged and complex disease do severely affects the physical and mental health of older patients and the ability of CDSM [34]. This study also discovered that older adults with low income and living alone had lower levels of CDSM, which is consistent with published findings [35]. These older adults often lack access to health information and have nobody to monitor and correct behaviors detrimental to disease management. And appropriate support is associated with better physical and psychological health outcomes and may enhance patients’ sense of responsibility to manage diseases [14]. Besides, patients from low-income households often worry about medical costs, creating a long-term psychological burden, and thus affecting self-management awareness and ability. Therefore, health education aimed at promoting chronic disease self-management should be targeted according to patients’ disease characteristics, living conditions, and economic status.

As we hypothesized, self-efficacy was positively associated with self-management in older patients with CNCDs. These findings agree with discoveries that imply self-efficacy has a positive effect on self-management in patients with type 2 diabetes, hypertension, and stroke [36, 37]. People with high self-efficacy also perceive their ability to overcome barriers and achieve goals such as diet modification, medication adherence, and disease management [15], and they can view the treatment process with better confidence and take more action to relieve loss and helplessness caused by the diseases, improving their self-management and forming a virtuous cycle. Our study also showed that self-efficacy mediates the association between e-health literacy and self-management behaviors, consistent with previous investigations [38]. Therefore, we recommended that health clinicians regularly assess older adults with CNCDs to understand their confidence in managing the condition. In this way, the patient’s self-efficacy should improve for enhanced chronic disease management ability.

Our study showed that e-health literacy is associated with CDSM, and this association is mediated by self-efficacy. E-health literacy score is within lower levels according to the international definition (total score < 26) [17]. It is similar to the one obtained for Chinese older adults [39] and lower than for American [40]. A systematic review concluded that internet-based interventions motivate patients to sustain health management and improve aspects of objective body indicators (systolic blood pressure, body-mass index, etc.), psychosocial dimensions (e.g., anxiety, depression), and other factors [41]. In the transition to a digital world, e-health facilitates older adults to obtain information about their illnesses. However, those with low e-health literacy may be unable to read and understand these information, rendering them unable or unwilling to use e-health resources. Healthcare professionals and policymakers should focus on training and improving e-health literacy of the older population to ensure that smart health products and internet services are utilized in practice. Furthermore, self-efficacy should be taken into account when using e-health information to enhance older patient’s CDSM.

Smart medical devices were positively correlated with CDSM in older adults, which agrees with previous findings [42]. The development and growth of these products have shown exciting potential and application in CDSM of older people due to convenient communication platforms and personalized health support. Smart medical devices help identify disease problems in a comprehensive and timely manner, enhancing medication adherence and maintaining a positive lifestyle [43]. Univariable analysis showed that products recommended by medical staff received higher SUS, uMARS, and product grade scores, which confirmed by previous research [44]. These data indicate that medical professionals can give personalized advice and specialized guidance to increase product use and benefit rate. In addition, smart medical devices for the older population should focus on simplicity of operation, functional variety, efficiency, and practicality. Hence, further empirical research is necessary to promote the standardization and functionality of medical devices. Taken together, our findings provide more reference for the development of smart medical devices and their promotion and application in elderly patients with chronic non-communicable diseases.

## Conclusions

Our study explored several factors (e-health literacy, self-efficacy, social support, and the application of smart medical devices) affecting CDSM of older patients with CNCDs. The structural equation model demonstrated the mediating role of self-efficacy between chronic disease self-management and e-health literacy, confirming our hypothesis. Future interventions should focus on improving e-health literacy, self-efficacy, and the utilization of smart healthcare devices to directly or indirectly benefit the self-management of patients with CNCDs.

## Limitations

Our study has certain limitations. First, it was cross-sectional, so the causal nature of influencing factors was not determined. A prospective design should be employed to investigate this question further. Second, the study was geographically restricted to Hunan Province, China, limiting generalization. Additionally, the study included only older adults who volunteered to participate, causing selection bias and relatively unrepresentative sample size. Future research should investigate a large, preferably global sample, allowing for a more detailed examination of self-efficacy and e-health literacy models of chronic disease management.

## Supplementary Information

**Additional file 1: Table S1.** Univariate analysis of general information and main study variables in older people with CNCDs (*n* = 289).

## Data Availability

The datasets used and analyzed during the current study are available from the corresponding author on reasonable request.

## Abbreviations

CNCDs: Chronic non-communicable diseases
CDSM: Chronic disease self-management
e-Health: electronic health
CCM: Chronic Care Model
ICCC: Innovative Care for Chronic Conditions Framework
WHO: World Health Organization
SUS: System Usability Scale
uMARS: User Version of the Mobile Application Rating Scale
