# Supplementary material for: Associations between e-health literacy and chronic disease self-management in older Chinese patients with chronic non-communicable diseases: a mediation analysis
Source: BMC Public Health. 2022 Nov 29;22:2226. doi: 10.1186/s12889-022-14695-4 (PMC9710041; doi:10.1186/s12889-022-14695-4)
Supplement: Supplementary file 1 — Additional file 1: Table S1. Univariate analysis of general information and main study variables in older people with CNCDs (n = 289). [file 12889_2022_14695_MOESM1_ESM.docx]

**Table S1.** **Univariate analysis of general information and** **main study variables in older people with CNCDs (*n* = 289)**

| Variable | N (%) | CDSM | | e-health literacy | | self-efficacy | | social support | |
| --- | --- | --- | --- | --- | --- | --- | --- | --- | --- |
|  |  | **±*s* | *t/F，P* | **±*s* | *t/F，P* | **±*s* | *t/F，P* | **±*s* | *t/F，P* |
| **Socio-demographic information** | | | | | | | | | |
| Gender |  |  |  |  |  |  |  |  |  |
| Male | 181 (62.6) | 28.84±9.09 | t=-1.273 | 18.40±9.41 | t=-1.723 | 24.39±6.45 | t=-1.186 | 41.81±5.84 | t=-1.539 |
| Female | 108 (37.4) | 30.32±10.37 | *P=*0.204 | 20.41±9.82 | *P=*0.086 | 25.32±6.59 | *P=*0.237 | 42.98±6.96 | *P=*0.125 |
| Age (years) |  |  |  |  |  |  |  |  |  |
| 60-70 | 185 (64.0) | 30.50±9.80 | F=3.093 | 25.47±5.62 | F=8.054 | 24.84±5.26 | F=3.159 | 43.63±4.49 | F=5.107 |
| 71-80 | 97 (33.6) | 27.85±9.47 | *P=*0.027 | 20.07±9.83 | P﹤0.001 | 25.15±6.26 | *P=*0.025 | 43.14±5.71 | *P=*0.002 |
| 81-90 | 7 ( 2.4 ) | 22.00±6.03 |  | 17.09±9.08 |  | 24.53±7.00 |  | 40.86±7.08 |  |
| Number of children | |  |  |  |  |  |  |  |  |
| 0 | 7 ( 2.4 ) | 33.00±10.74 | F=1.264 | 21.00±10.54 | F=0.643 | 29.00±7.66 | F=4.760 | 43.43±5.80 | F=0.327 |
| 1 | 49 (17.0) | 30.83±9.64 | *P=*0.284 | 20.37±9.61 | *P=*0.526 | 26.73±6.57 | *P=*0.009 | 42.73±6.82 | *P=*0.722 |
| ≥2 | 233 (80.6) | 28.99±9.85 |  | 18.84±9.59 |  | 24.19±6.36 |  | 42.10±6.21 |  |
| Marital status | |  |  |  |  |  |  |  |  |
| Married | 182 (63.0) | 30.51±9.26 | F=4.276 | 20.32±9.32 | F=5.054 | 25.68±6.39 | F=4.078 | 43.25±6.01 | F=11.633 |
| Single | 62 (21.5) | 30.15±10.02 | *P=*0.006 | 18.68±10.21 | *P=*0.002 | 23.76±6.30 | *P=*0.007 | 42.23±4.63 | *P<*0.001 |
| Widowed | 36 (12.5) | 24.72±8.86 |  | 13.72±8.26 |  | 22.03±6.87 |  | 36.86±7.66 |  |
| Divorced | 9 ( 3.1 ) | 25.00±29.39 |  | 20.44±9.42 |  | 23.33±5.39 |  | 43.56±5.50 |  |
| Education status | |  |  |  |  |  |  |  |  |
| Primary school | 97 (33.6) | 28.13±8.74 | F=1.082 | 13.67±7.33 | F=19.330 | 22.01±6.16 | F=10.901 | 39.60±6.91 | F=11.089 |
| Junior school | 96 (33.2) | 30.39±10.40 | *P=*0.357 | 21.40±9.29 | *P<*0.001 | 25.20±6.45 | *P<*0.001 | 43.19±5.00 | *P<*0.001 |
| High school | 66 (22.8) | 29.23±9.21 |  | 21.98±9.68 |  | 27.23±5.52 |  | 44.79±5.02 |  |
| University | 30 (10.4) | 30.67±10.37 |  | 23.46±9.49 |  | 26.60±6.87 |  | 42.20±7.46 |  |
| Occupation | |  |  |  |  |  |  |  |  |
| Farmer | 85 (29.4) | 28.54±7.99 | F=6.273 | 15.32±8.43 | F=6.047 | 22.34±6.66 | F=5.710 | 41.02±7.29 | F=1.604 |
| Self-employed | 33 (11.4) | 36.82±11.03 | *P<*0.001 | 23.20±6.44 | *P<*0.001 | 27.30±6.58 | *P<*0.001 | 42.94±5.73 | *P=*0.173 |
| Company staff | 10 ( 3.5 ) | 26.30±6.23 |  | 22.88±7.22 |  | 24.20±4.28 |  | 43.70±4.62 |  |
| Public staff | 12 ( 4.2 ) | 16.50±6.53 |  | 21.67±9.33 |  | 28.33±6.05 |  | 40.50±6.41 |  |
| Retired | 149 (51.6) | 28.68±9.83 |  | 20.02±9.61 |  | 25.28±6.15 |  | 42.83±5.80 |  |
| Living situation | | | | | | | | | |
| Alone | 97 (33.6) | 28.33±9.60 | F=2.877 | 16.55±9.49 | F=6.883 | 22.89±6.48 | F=4.357 | 40.40±6.37 | F=5.157 |
| With spouse | 88 (30.4) | 31.49±9.30 | *P=*0.036 | 22.27±9.15 | *P<*0.001 | 25.49±6.56 | *P=*0.005 | 43.41±5.17 | *P=*0.002 |
| With child | 34 (11.8) | 30.71±12.43 |  | 21.26±10.71 |  | 26.59±5.83 |  | 41.71±7.33 |  |
| With family | 70 (24.2) | 27.60±7.86 |  | 17.81±8.54 |  | 25.46±6.32 |  | 43.60±6.41 |  |
| Residence time (years) | |  |  |  |  |  |  |  |  |
| <5 | 100 (34.6) | 27.05±10.41 | F=4.838 | 17.61±9.71 | F=2.031 | 24.96±6.85 | F=0.166 | 41.56±6.80 | F=2.202 |
| 5-10 | 12 ( 4.2 ) | 29.17±7.48 | *P=*0.009 | 20.75±8.80 | *P=*0.133 | 25.33±6.56 | *P=*0.847 | 45.42±8.82 | *P=*0.112 |
| ≥10 | 177 (61.2) | 30.73±9.02 |  | 19.92±9.53 |  | 24.57±6.34 |  | 42.42±5.74 |  |
| Main caregiver | |  |  |  |  |  |  |  |  |
| Own | 58 (20.1) | 27.76±9.74 | F=1.514 | 18.12±10.74 | F=1.244 | 27.19±6.89 | F=3.866 | 43.71±7.39 | F=4.813 |
| Spouse | 114 (39.4) | 30.80±9.49 | *P=*0.211 | 20.48±9.75 | *P=*0.294 | 24.54±6.30 | *P=*0.010 | 43.20±4.35 | *P=*0.003 |
| Child | 107 (37.0) | 28.83±9.66 |  | 18.29±8.82 |  | 23.70±6.21 |  | 40.62±6.81 |  |
| Caregiver | 10 ( 3.5 ) | 28.90±8.48 |  | 19.20±8.51 |  | 23.80±6.96 |  | 40.30±8.74 |  |
| Monthly income (RMB) | |  |  |  |  |  |  |  |  |
| <2000 | 126 (43.6) | 26.63±8.12 | F=16.081 | 16.01±8.62 | F=13.027 | 21.94±6.05 | F=23.999 | 40.87±6.33 | F=5.524 |
| 2000-5000 | 101 (34.9) | 33.45±10.27 | *P<*0.001 | 21.74±9.95 | *P<*0.001 | 26.90±5.83 | *P<*0.001 | 43.20±5.75 | *P=*0.004 |
| ≥5000 | 62 (21.5) | 28.42±9.10 |  | 21.32±9.17 |  | 26.90±6.36 |  | 43.48±6.60 |  |
| **Health-related information** | | | | | | | | | |
| Hospitalization history | |  |  |  |  |  |  |  |  |
| Yes | 224 (77.5) | 28.79±9.48 | t=1.982 | 18.51±9.50 | t*=*2.128 | 24.33±6.40 | t=1.982 | 42.20±5.82 | t=0.224 |
| No | 65 (22.5) | 31.46±9.79 | *P=*0.048 | 21.37±9.67 | *P=*0.034 | 26.14±6.73 | *P=*0.048 | 42.40±7.76 | *P=*0.823 |
| Family history | |  |  |  |  |  |  |  |  |
| Yes | 67 (23.2) | 29.85±9.92 | t=-0.443 | 19.15±8.90 | t*=*0.003 | 24.61±6.73 | t=0.179 | 40.70±7.36 | t=2.309 |
| No | 222 (76.8) | 29.26±9.52 | *P=*0.658 | 19.15±9.82 | *P=*0.998 | 24.77±6.45 | *P=*0.858 | 42.71±5.87 | *P=*0.022 |
| Years of CNCDs | |  |  |  |  |  |  |  |  |
| <5 | 60 (20.8) | 32.58±10.64 | F=5.979 | 20.45±10.42 | F=5.278 | 27.12±6.70 | F=8.707 | 43.42±6.00 | F=1.871 |
| 5-10 | 85 (29.4) | 30.04±9.60 | *P=*0.003 | 21.27±9.73 | *P=*0.006 | 25.53±5.81 | *P<*0.001 | 42.51±5.94 | *P=*0.156 |
| >10 | 144 (49.8) | 27.69±8.78 |  | 17.36±8.87 |  | 23.28±6.48 |  | 41.60±6.57 |  |
| Number of CNCDs | |  |  |  |  |  |  |  |  |
| 1 | 63 (21.8) | 32.08±10.79 | F=4.383 | 21.05±10.34 | F=1.936 | 26.60±6.93 | F=11.888 | 43.10±6.56 | F=2.521 |
| 2 | 134 (46.4) | 27.85±8.61 | *P=*0.013 | 18.18±9.26 | *P=*0.146 | 22.81±5.77 | *P<*0.001 | 41.36±6.03 | *P=*0.082 |
| ≥3 | 92 (31.8) | 29.80±9.76 |  | 19.27±9.46 |  | 26.27±6.50 |  | 42.96±6.37 |  |
| Frequency of medication | |  |  |  |  |  |  |  |  |
| 1 | 26 ( 9.0 ) | 33.00±12.01 | F=2.449 | 23.23±9.90 | F=3.255 | 27.19±6.98 | F=3.367 | 43.23±7.11 | F=0.803 |
| 2 | 13 ( 4.5 ) | 26.69±8.72 | *P=*0.088 | 15.84±8.19 | *P=*0.040 | 27.31±6.36 | *P=*0.036 | 43.77±7.45 | *P=*0.449 |
| ≥3 | 233 (80.6) | 18.84±9.59 |  | 42.10±6.21 |  | 24.19±6.36 |  | 28.99±9.85 |  |
| Use smart health devices | |  |  |  |  |  |  |  |  |
| Yes | 46 (15.9) | 33.43±11.66 | t=9.999 | 24.63±9.84 | t=18.929 | 27.80±6.74 | t=12.651 | 45.35±5.86 | t=13.894 |
| No | 243 (84.1) | 28.63±8.98 | *P=*0.002 | 18.12±9.21 | *P<*0.001 | 24.16±6.31 | *P<*0.001 | 41.66±6.21 | *P<*0.001 |

**±s: Mean ± standard deviation, CDSM: chronic disease self-management.
